# The dynamic bodily self across lifespan: Agency, ownership, and the plasticity of internal body models

**DOI:** 10.3758/s13423-026-03024-0

**Published:** 2026-10-09

**Authors:** Amir Jahanian Najafabadi, Matthew R. Longo, Roman Liepelt

**Affiliations:** 1 Department of Cognitive Neuroscience, Bielefeld University, Bielefeld, Germany; 2 School of Psychological Sciences, Birkbeck University of London, London, UK; 3 Department of General Psychology: Judgment, Decision Making, Action, Faculty of Psychology, FernUniversität in Hagen, Hagen, Germany

**Keywords:** Body ownership, Agency, Rubber hand illusion, Body representation, Consciousness, Internal body models, Aging, Life span

## Abstract

Two fundamental aspects of research on bodily self-consciousness, agency and body ownership, have recently seen exponential growth. The seminal paper of Botvinick and Cohen in 1998 provided a vital psychophysical tool for studying the bodily self – the rubber hand illusion, in which one feels as if an external object becomes a part of one’s body. Recent motor control views of ownership highlight the influence of agency, the sense that one is in control of one’s movements. Technological advancements have facilitated more ecologically valid experimental paradigms, such as virtual versions of the rubber hand illusion, furthering our understanding of the mechanisms through which the brain generates agency and ownership sensations. Recent findings suggest that agency and ownership can interact while remaining dissociable, as one may experience agency over an object that is not embodied or ownership of an object that cannot be controlled. Understanding the neurocognitive mechanisms underlying agency and ownership is critical for addressing the challenges of healthy aging. As sensory-motor precision declines across the lifespan, maintaining a coherent sense of body and control becomes essential for mobility, the successful adoption of assistive technologies, and overall cognitive resilience. This review synthesizes current findings on the agency-ownership interaction, discusses its neurobiological substrates, and explores its transformative potential for the design of “self-inclusive” prosthetics and brain-machine interfaces. We conclude by outlining a framework showing how optimization of these processes can support functional independence and psychological well-being in an aging population.

**Supplementary Information:**

The online version contains supplementary material available at https://doi.org/10.3758/s13423-026-03024-0.

## Introduction

The concept of the self is central to understanding human identity, behavior, and consciousness. It can be thought of as a bundle of perceptions (sensations, thoughts, and feelings) (Hume, 1739) or traits that define the self. The self emerges as a complex system, shaped by the dynamic interactions of its various components such as social self, embodied self, minimal self, narrative self, extended self, and ecological self (Gallagher, 2013). Over the years, researchers from many fields such as philosophers, psychologists, neuroscientists, and roboticists have explored various dimensions of the self, leading to different interpretations and models of what it means to be a self (for review, see Blanke & Metzinger, 2009; Cross & Ramsey, 2021; Forch & Hamker, 2021; Gallagher, 2000; Prescott et al., 2024). The self reflects a combination of both our internal self-perception and the way in which others perceive us, and it is not a single, fixed entity but rather a dynamic and multifaceted concept composed of several interrelated components (see Sparby et al., 2019; Woźniak, 2018). Overall, these parts of the self are not separate or isolated, they constantly influence and overlap with one another. According to Gallagher, it is through this ongoing dynamic interaction that our sense of self takes shape, making it complex and dependent on our situational context.

Gallagher (2000) initially distinguished between two aspects of the self: (1) The minimal self, which refers to our immediate, present moment sense of self – the “I” that perceives and experiences in the current moment. This immediate aspect of the self is defined by key components that anchor us to our present reality: *body representations* (our real-time perception of our body), *body ownership* (the feeling that this body is mine), and *sense of agency* (the feeling that I am the one controlling my actions). (2) Another aspect of the self is the *narrative self,* which encompasses a broader, time-spanning understanding of ourselves. The narrative self includes stories we construct about our lives and identity, often shaped by social interactions and experiences. As suggested by Gallagher and Daly (2018), narrative elements might involve (a) repeatedly revising conversations that felt unresolved or unsatisfactory; (b) frequent use of first-person pronouns; (c) past narratives focusing on themes of loss, failure, or harm; (d) present narratives showing minimal engagement or interest; and (e) future narratives appearing absent or lacking development.

According to pattern theory (Gallagher, 2013), the self is not seen as a single, fixed entity but is instead understood as a dynamic system of interrelated dimensions (embodied, affective, social, narrative, and extended technological and cultural aspects). These dimensions continuously influence each other, forming an integrated, complex experience that we recognize as the self. Based on this integrated multidimensional structure, the self is not static, but rather a dynamically evolving formation.

## Minimal self, movement, and agency

The minimal self is a crucial dimension directly linked to our immediate, lived experience, especially in relation to action and movement. It is intimately connected with the motor aspects of experience, i.e., the planning, execution, and perception of bodily movements. This link forms the basis of two essential perceptions: body ownership, the feeling that the moving body belongs to oneself, and the sense of agency, the feeling that I am the one initiating and controlling the movement (De Vignemont, 2010; Gallagher, 2000). These perceptions are not abstract constructions; they rely on ongoing movement and sensory feedback. For example, smoothly controlling one’s arm enhances both the sense that the arm belongs to oneself and the sense that one is acting intentionally.

The motor aspect of the minimal self explains how the self develops through our ongoing interaction with the environment, emphasizing the constant relationship between what we perceive, what we do, and what we expect to happen (Nguyen et al., 2021). Perception gives us sensory information about our body and our environmental context. Action involves carrying out movements in response to our perceptions, and prediction reflects the brain’s attempt to anticipate the results of those movements before they actually occur. When what we predict lines up with the sensory feedback we actually experience as a direct consequence of our actions, the brain successfully matches the intended motor command with the resulting environmental change, and our sense of body ownership and agency becomes stronger. In this way, the cognitive and motor processes allow the minimal self to form and remain relatively stable over time.

Having a clear view that a hand that is perceived as belonging to my body, which can be differentiated from other people’s hands or objects of the external world, for example, makes tools appear relatively easy. However, patients with neurological disorders or amputees who have replaced a lost body part with a prosthesis show that it is much less clear under which conditions one perceives an object as part of their bodily self. In this conceptual and theoretical review, we will discuss the mechanisms underlying body ownership with a special focus on the functionality of our body and the sense of agency, the feeling of being the owner of an action (Caspar et al., 2015; De Vignemont, 2007; Ma & Hommel, 2015a; Rognini et al., 2013). In particular, we synthesize evidence on the mechanisms underlying body ownership and agency, exploring their dynamic interplay and extending this framework to understand lifespan plasticity, particularly in the context of healthy aging, motor learning, and the integration of assistive technologies. The following section discusses the association between a subjective sense of ownership and agency with the plasticity of body schema and body image, which remains under debate, through the lifespan, particularly studied during sequential motor learning tasks. The understanding of the mechanisms that mediate bodily self-perception is partly due to recent theoretical advancements, in conjunction with the development of innovative paradigms, new technological devices like virtual reality (Lenggenhager et al., 2007; Slater et al., 2010), and the need for improved integration in robots and robotic prosthetics.

The self is subjectively often perceived as relatively stable and constant over time. However, research on patients and healthy subjects shows that the minimal self is much more plastic than this subjective intuition suggests (Longo & Haggard, 2012). Experimentally, it is remarkably easy to produce disturbances of the bodily self to incorporate an external object (Kilteni et al., 2015; Slater et al., 2010) like a rubber hand (Botvinick & Cohen, 1998), a virtual avatar (Slater et al., 2010), or even other people’s bodies (Lenggenhager et al., 2007). The malleability of the bodily self allows the body to become functionally integrated with the external world, extending the ways in which we can act upon the environment (e.g., when controlling an excavator).

## Emerging methods to measure bodily self-perception

One of the first studies of bodily illusions suggested a role of sensory feedback for constructing a body image by showing that people often misjudge a plastic finger from underneath a cloth as their real finger (Tastevin, 1937). Merleau-Ponty (1945/2012) described self-recognition as being more than the immediate experience of having a feeling of our body. It also involves the visual perception of our bodily self, in a similar way to how we perceive others. In the rubber hand illusion (RHI; Botvinick & Cohen, 1998), the experimenter strokes the subject’s arm, which is hidden from view, and a rubber hand that is placed next to the real arm. The stroking of the rubber hand and the real arm is done temporally synchronously or asynchronously by using two separate paintbrushes. The rubber hand illusion produces three distinct effects consistent with the notion of Merleau-Ponty: the misattribution of ownership to an external object, a shift in perceived position of the arm, and the mislocalization of touch (Tsakiris et al., 2010).

After the induction of the illusion, subjects typically respond to a series of questions related to body ownership on a Likert or analogous scale (Kalckert & Ehrsson, 2012, 2014). This survey tests the core aspect of the RHI, that subjects report a feeling that the rubber hand feels like their real hand after synchronous but not after asynchronous stroking. In contrast, *proprioceptive drift* reflects an implicit measure of the effects of the RHI on body ownership. Subjects are asked to indicate where they perceived their hand to be after synchronous or asynchronous stroking by reading off the number on a ruler. After synchronous stroking, they are more biased to point towards the rubber hand, pointing to the space between the rubber hand and their real hand, suggesting they had embodied the seen object as part of their own body. It remains contentious as to whether the ownership ratings and the proprioceptive drift measures reflect different aspects of ownership, with studies even showing there is no causal link between these measures (e.g., Abdulkarim & Ehrsson, 2016).

Over recent decades, different groups have tested body ownership, agency experiences, and emotional reactions to the threat of the virtual body. Several studies have examined whether threats directed at an embodied rubber hand elicit physiological responses similar to those observed when the subject’s real hand is threatened. Such responses have served as an indicator of whether the rubber hand is perceived as a part of the body. Armel and Ramachandran (2003) observed an increase in sweat production, known as the *skin conductive response*, when they bent a finger on the rubber hand into a normally excruciatingly painful position. In line with this, Ehrsson et al. (2007) showed that brain areas related to anxiety and interoceptive awareness (insula and anterior cingulate cortex) show similar levels of brain activity when threatening an embodied rubber hand and the real hand of the subjects. Showing such correlations between reports about the RHI and the strength of physiological responses to threat indicates that the illusion has a clear correspondence in the neural system relevant for regulating bodily defence responses.

Moreover, in the study conducted by Cardinali et al. (2021), researchers investigated body ownership by examining how individuals respond to an arm-shaped mechanical grabber used as an extension of the body. Their findings revealed that participants exhibited an altered skin conductance response when a potentially threatening stimulus approached the mechanical grabber.

Complementary research by Rossetti et al. (2015) focused on how intensive tool-use training determines the plasticity of peripersonal space, the immediate defensive and action-oriented zone surrounding the body. Their findings indicated that proficiency gained through extended tool use resulted in a substantial expansion of peripersonal space, a change quantified by the skin conductance response metric. Post-training, the distance at which an approaching object triggered a significant increase in skin conductance (i.e., signalling that the stimulus had entered the perceived threat zone) was measured to be substantially farther from the participant’s torso. This physiological change reflects a highly adaptive neural recalibration: the peripersonal space is temporarily remapped to encompass the tool’s extended reach, thus establishing the tool’s boundary as the new frontier for potential threat and requiring the brain to adjust its entire spatial defensive mapping. This physiological response, typically linked to stress or heightened arousal, was interpreted as evidence that participants experienced the grabber as part of their own body rather than a distinct object. The results suggest that the brain’s representation of the body is flexible enough to incorporate external tools when they are used in ways that resemble ordinary bodily actions. This flexibility highlights how body ownership relies on the integration of multiple sensory signals. Visual information, touch, and motor feedback work together to generate a coherent sense of the body as a unified whole. When these cues align, external devices can come to feel as though they belong to the user.

These findings are particularly relevant for understanding how humans interact with tools and emerging technologies. In areas such as prosthetics, robotics, and virtual reality, strengthening the feeling of body ownership over external devices may improve motor performance and enhance user experience. The more a tool becomes integrated into the body representations, the more movements become more intuitive, learning progresses more efficiently, and the cognitive effort required to control the device decreases. A strong sense of ownership also appears to support accurate motor prediction. The brain can generate efference copies more reliably and reduce prediction errors when the tool is perceived as “self.” This leads to a stronger sense of agency, allowing control to become smooth, automatic, and effortless rather than consciously managed.

The flexibility of body ownership allows external devices to become embodied, a process wherein an external effector is integrated into bodily self-representation involving shifts in ownership, agency, and sensorimotor body representations (Martel et al., 2016; Weser & Proffitt, 2021). When a device moves like a real limb or responds to our intentions, the brain can start to treat it as a part of the body. This ability to incorporate external objects into our sense of self plays a key role in how comfortably and effectively we interact with technologies like prosthetics or robotics for lower limbs such as exoskeletons (see Hybart & Ferris, 2023) and semi-autonomous control of prosthetic hands (Starke et al., 2022). Embodiment has now further received significant attention in robotic assistive technology and immersive metaverse, which enables us to interact in a simulated digital environment that incorporates augmented, virtual, and extended realities (Beckerle et al., 2018; Riva & Wiederhold, 2022).

The RHI has also been extended to the full body (Lenggenhager et al., 2007), where the illusion changes not just what objects are part of the body, but what body the self is located within. Stroking of the real body in synchrony with visual stimulation of a manikin produces multisensory correlations resulting in illusory ownership over the fake body and the feeling of disembodiment of the real body (Salomon et al., 2013). Slater et al. (2010) demonstrated a body transfer illusion for male subjects into a virtual human female body, measured by subjective questionnaires and physiological responses measured through *heart-rate* deceleration. The latter studies show that the induction of body ownership illusions does have physiological consequences for the real body.

For a more comprehensive understanding of the specific components that make the illusion in the RHI paradigm possible, researchers utilized the same paradigm, but in various experimental settings. This approach helps them to look for an answer to specific questions regarding: (1) The extent of the influence of the incongruent direction of stroke (Costantini & Haggard, 2007); (2) the extent of the change in the strength of the illusion if the fake hand was placed in the spatially incongruent position at a different location (Armel & Ramachandran, 2003; Llyod, 2007) or orientation (Guterstam et al., 2011; Pavani et al., 2000; Tsakiris & Haggard, 2005); and (3) the degree of the influence of the visual appearance of the fake hand on the emergence of the illusion. The similarity or difference in visual appearance between fake and real hands can include different aspects, such as anatomical shape (Tsakiris et al., 2010), laterality (Petkova & Ehrsson, 2008), and size (Pavani & Zampini, 2007).

A key challenge arising from the application of the RHI in experimental studies is that most studies using this paradigm rely primarily on explicit, subjective measures of body ownership, such as questionnaires given after the RHI session. However, implicit measures such as the proprioceptive drift of the perceived location of the real hand may provide a more sensitive and objective way of evaluating the body ownership update and the occurrence of the illusion (Longo et al., 2008; Tsakiris & Haggard, 2005). However, the validity of the proprioceptive drift as a standalone proxy for ownership has been criticized. Reviews by Rohde et al. (2011) suggest that the proprioceptive drift may arise even when subjects do not consciously experience the illusion of ownership. This finding indicates that proprioceptive drift may reflect a low-level sensory spatial recalibration rather than the higher-order cognitive integration of an external object into the self-model. Consequently, modern experimental designs are increasingly moving toward a multidimensional approach that triangulates explicit reports, behavioral drift, and physiological data to capture a more holistic picture of the embodied self.

## Theoretical grounding of bodily self-perception: The sense of ownership

The sense of ownership refers to a unique perceptual experience in which an individual recognizes their body, sensations, or thoughts as inherently belonging to themselves, a sense often described as “mineness.” This feeling of ownership reflects the fundamental awareness that “my body” is distinctly “mine,” setting it apart from external objects or the bodies of others. The concept of body ownership has been described in various ways, reflecting its multifaceted nature. Schütz-Bosbach and colleagues (2009) referred to it as the “phenomenological sensation of incorporation,” emphasizing the subjective experience of feeling one’s body as a cohesive whole.

Similarly, Butler and colleagues (2017) framed it as the ability to know “which body parts belong to us,” highlighting its role in self-identification. Others, such as Berlucchi and Aglioti (1997), have used the term body ownership interchangeably with “corporeal awareness,” underlining its connection to physical self-recognition. Longo and colleagues (2008) succinctly captured the complexity of studying body ownership, noting, “It is clearly a kind of experience, but psychology’s traditional methods of studying experience have difficulty in capturing its nature.” This suggests that understanding body ownership challenges conventional approaches to examining subjective phenomena, necessitating innovative methods to fully explore its underlying mechanisms and implications.

Braun et al. (2018) and Tsakiris (2010, 2016) have emphasized that ownership arises from a perceptual distinction that is closely tied to the subjective experience of selfhood. This perceptual distinction refers to the way the brain differentiates between what is perceived as part of oneself (the bodily self) and what is perceived as external or separate from the self (like other bodies or objects), highlighting the sensory and cognitive process that enables a person to recognize their body or experiences as their own (Chancel & Ehrsson, 2020; Tsakiris, 2016). This perceptual process enables a person to recognize specific body parts or experiences as uniquely their own; it is dynamic, depending on contextual factors and personal engagement, which is linked to the self-concept (Baumeister, 1998; Oyserman et al., 2012). It manifests strongly in conditions where one feels a direct connection to one’s body and may diminish or be absent in situations where such a connection is disrupted. For instance, the sensation of ownership is profoundly evident during the execution of movements, regardless of whether they are performed voluntarily or involuntarily. As Gallagher (2000) noted, the sense of ownership is tied to the implicit understanding that “I am the one [experiencing]” these bodily actions or sensations. Moreover, the concept of ownership is critical in distinguishing one’s body from external entities. It allows individuals to perceive their limbs, sensations, and movements as uniquely their own, even in complex scenarios such as interacting with external tools or adapting to new environments.

Some theoretical models attributed the sense of ownership in the RHI to the multisensory integration of signals coming from vision, touch, and proprioception (Armel & Ramachandran, 2003; Makin et al., 2008). According to this *multisensory integration model*, in principle different sorts of objects may be integrated in one’s body perception, as long as signals from two or more modalities are synchronized. Armel and Ramachandran (2003) describe body integration as a Bayesian binding process. For instance, two signals coming from vision and touch that are temporally co-occurring with high probability are interpreted to arise from one object. The notion is that the brain detects pure statistical correlations in different sensory inputs and uses these inputs to construct useful conceptual representations out of these sensory inputs (Armel & Ramachandran, 2003).

The neurocognitive model of body ownership builds upon the multisensory integration model by assuming that body ownership is thought to emerge from the integration of ongoing multisensory inputs including visual and tactile signals with pre-existing neural representations of the body and the minimal self (Tsakiris et al., 2010). The model proposes a comparison along three steps. The first step distinguishes between objects that may be part of one’s body and objects that do not belong to the body, by comparison with a pre-existing internally stored minimal self-model that includes anatomical and structural descriptions of the body. In the second step, online representations of anatomy and posture modulate the degree of multisensory integration, which can then elicit a recalibration of visual and tactile reference frames. In the third step, the updated tactile sensations contribute to the subjective experience of body ownership (Tsakiris, 2010). This model is consistent with the brain using stored knowledge to optimize incoming sensory processing of events and to derive predictions to remove the prediction error in multisensory body representations when seeing and feeling a touch at different locations (Apps & Tsakiris, 2013; Ferri et al., 2017; Friston, 2010). Within this predictive coding framework, body ownership and recognition are the functional outcomes of the brain explaining incoming sensory information using the most parsimonious or efficient internal model, a concept known as the “free-energy self.” The system operates through a hierarchical organization where top-down contextual information modulates the processing of bottom-up sensory “surprise” or prediction errors. Crucially, research by Apps and Tsakiris (2013, 2014) using functional magnetic resonance imaging (fMRI), identified that this perceptual learning process relies on the continuous updating of stimulus familiarity independently of the specific viewpoint. For instance, visual face recognition is heavily influenced by top-down expectations about likely sensory input processed prior to the stimulus itself, which is independent of the specific visual properties of the stimulus. The final decision to recognize or embody a stimulus is a function of both the learned stimulus properties and current contextual expectations to guide behavioral choices and decisions. By integrating these predictive mechanisms, the neurocognitive system can resolve the spatial and temporal conflicts inherent in multisensory body representations, ensuring that the minimal self is maintained through an efficient, adaptive balance between stored prior knowledge and dynamic environmental feedback.

## The sense of agency

The sense of agency refers to the feeling of being the initiator of one’s actions (De Vignemont, 2007; Gallagher, 2000, 2018; Ma & Hommel, 2015a). The sense of agency refers to the pre-reflective and intuitive sense that one is the originator of a movement or action, whether it is carried out voluntarily or involuntarily. It encompasses the subjective experience of being in control of one’s own actions and their consequences. For example, when reaching to grasp a cup or inadvertently flinching upon a loud noise, individuals often perceive themselves as the source of these movements without the need for conscious reflection. The sense of agency seems fundamental for the understanding of how people attribute actions to themselves and how they distinguish their own actions from external influences or other people’s actions. As highlighted by Tsakiris et al. (2010), the sense of agency also plays a critical role in human cognition and self-perception. It underpins the sense of autonomy and ownership over one’s actions, forming a foundation for interactions with the external world. Moreover, it is closely tied to motor control processes and sensory feedback mechanisms, which continuously inform the brain about the outcomes of one’s movements. This intricate interplay allows individuals to align their intentions with executed actions, reinforcing their sense of being in control.

For example, it was suggested that delivering feedback to specific body parts during a motor task shifts external and internal focus of attention toward the goal, and movement coordination and body parts under training, respectively. Directing attention toward associated body parts enhances the sense of agency, leading to an improvement in motor performance. This notion was supported through our studies (Jahanian Najafabadi et al., 2023a, 2023b), demonstrating an improvement in practice effect during training combined with visuo-haptic feedback delivered to a real hand during virtual tool-use training in both young and older adults. Guiding learners to shift their attention from self or their body part towards the task goal and determined movement, and emphasizing on the production of specific task conceptions, enforce motor learning and performance (Wulf & Lewthwaite, 2016).

A theoretical review (Wulf & Lewthwaite, 2016) suggested that even a modest increase in opportunities for choice and self-determination within a task can enhance learners’ perceived autonomy and sense of agency. These enhanced perceptions may in turn contribute to improved motor performance and motor skill learning. Additionally, it was suggested that granting learners a minimal degree of control over their practice conditions (e.g., information delivery, timing of feedback, and practice scheduling, a concept known as self-controlled practice) significantly enhances the learning effect (Wulf & Lewthwaite, 2016). This means that allowing learners to make intentional, small decisions about their training environment, rather than strictly imposing every variable, results in better motor learning outcomes.

The importance of the sense of agency is also addressed in research that focuses on its immediate, subjective experience during voluntary action. This includes work by Haggard et al. (2002) that focused on measuring the conscious awareness of voluntary actions using the effect of intentional binding. Intentional binding is a phenomenon where the perceived temporal interval between an action and its sensory consequence is reduced, serving as an operational measure of the strength of the sense of agency over that action. Moreover, in recent studies we discussed the emergence of the sense of agency and its association with higher tool use performance levels in older adults (e.g., Jahanian Najafabadi et al., 2023b, 2025).

In this context, further results have revealed clear differences between young and older adults in how they experienced and controlled a virtual tool. In young adults, increased mental load strengthened their sense of ownership over the tool, while physical load reduced their sense of agency. These effects were less pronounced in the older group, where only performance load emerged as a reliable predictor, leading to a higher sense of agency during tool use. The data collected through the embedded analogue scale, namely a “virtual slider,” provided an insight into moment-by-moment perceptions of sense of control over the virtual tool. Participants who reported a stronger sense of control tended to perform tasks faster, regardless of age. In contrast, ownership over the virtual tool did not relate to performance speed. Agency alone also did not directly predict faster completion times, but its relationship with performance differed across the age groups, suggesting that young and older adults at least partly rely on different underlying processes when establishing a sense of control during tool use (Jahanian Najafabadi et al., 2025). This aligns with the concept of autonomy supportive effects (Wulf & Lewthwaite, 2016), which further suggests that enhanced autonomy and sense of agency improve self-efficacy, and consequently performance of subjects. Further, providing instructions by the experimenter or various types of feedback given during motor learning tasks can influence the perception of sense of agency and autonomy.

The relationship between agency and ownership is bi-directional and dynamic. A strong sense of agency over an external object (like a tool or prosthetic) increases the precision of action-related sensory feedback, thereby making the object a more reliable candidate for integration into the body schema and strengthening ownership (Kilteni et al., 2012). Conversely, the prior establishment of ownership (e.g., through visual-tactile synchrony) can increase the expectation, or prior belief, that the body part will move successfully, thereby enhancing the subsequent sense of agency over its movements (Braun et al., 2018).

There are several theoretical models to explain the sense of agency. One is a basic model of motor control, the *comparator model* (Frith et al., 2000a; Wolpert et al., 1995), which is related to the reafference principle of Von Holst and Mittelstaedt (1950). In the comparator model, the role of internal sensorimotor processes in shaping our sense of agency is emphasized, while the theory of apparent mental causation (Wegner, 2002; Wegner & Wheatley, 1999) focuses more on how we interpret external events to determine causality. The cue integration theory brings these ideas together, proposing that our sense of agency is formed by blending both internal signals (like predictions about our movements) and external information (see van der Wel & Knoblich, 2013). The influence of each type of cue can vary depending on the specific aspect of agency being considered. Generally, we first rely on internal predictions, which are then combined with outside cues to form a conscious judgment about whether we caused an action (Moore et al., 2009; Synofzik et al., 2008).

To differentiate between self-generated sensory stimuli and those stimuli that are externally generated, the actual sensory input is compared with a predicted sensory input that is based on a copy of the motor command (i.e., the efference copy; for review, see Davidson & Wolpert, 2005). When there is a match between the efference copy and the sensory input (i.e., the reafference), a sense of agency arises and the movement is perceived as self-caused (Sperry, 1950). When there is a mismatch between the efference copy and the sensory input, there is no sense of agency and the movement is externally attributed (Braun et al., 2018).

The classic comparator model has been extended to the *multifactorial two-step account of agency* (Synofzik et al., 2008), often integrated into a broader Predictive Processing framework. This framework postulates that the sense of agency is not solely based on the efference copy match, but rather on the dynamic integration of multiple cues, including intentional cues (prior intention to act), outcome cues (matching goals to results), and the basic sensorimotor prediction. Studies indicate that besides the reafference principle from motor control, depending on access and reliability of the information, other agency cues may also contribute to the sense of agency (Moore, 2016; Synofzik et al., 2008). This *multifactorial two-step account of agency* (Synofzik et al., 2008) assumes that the sense of agency is explained by a multifactorial weighting process of different agency cues (step 1), followed by a conceptual module forming an attribution judgment (step 2).

The relative influence of different agency cues is dynamically weighted according to their context-dependent reliability and combined through *Bayesian cue integration* (Braun et al., 2018; Synofzik et al., 2009). According to Bayesian cue integration (Moore & Fletcher, 2012), each agency cue contains uncertainty. To reduce this uncertainty, the available agency cues are integrated according to maximum-likelihood principles (i.e., cue integration), producing a reduced signal variance as compared to each individual agency cue itself (Braun et al., 2018; Moore & Fletcher, 2012; Moore et al., 2009). This process relies on the brain continuously assigning a precision weight (or inverse variance) to each cue based on the cue’s reliability under specific contextual conditions. Highly precise cues (e.g., strong visual confirmation or a clear motor intention) are weighted more heavily in the final judgment of agency. The integration of multisensory information in the brain seems to follow a maximum likelihood estimation, which also makes Bayesian cue integration a likely mechanism for agency inference (Braun et al., 2018; van Dam et al., 2014). Cue integration based on a maximum-likelihood estimation combines agency cues through a weighting that is based on the reliability of such cues. As a Bayesian approach is used for cue integration, prior beliefs and belief updating can also be realised, increasing the robustness of the sense of agency over time (for review, see Franklin & Wolpert, 2011).

This Bayesian perspective is particularly powerful for modelling lifespan plasticity and technology integration because it provides a computational framework for describing how the brain constantly re-evaluates and combines sensory information to form a coherent self. The brain combines information from different senses and movement signals, giving more attention to the cues it considers most accurate. When someone ages or starts using a prosthetic device, some internal signals like knowing where the limb is or predicting how it will move can become less reliable. In these situations, the brain shifts its strategy. It places less weight on those internal bodily signals and instead depends more on what can be seen or on the visible results of the action (the actual visual sensory feedback). This shift helps maintain a stable sense of control over movement even when the body’s usual signals are not fully precise. The use of assistive technologies or the age-related decline in proprioceptive accuracy necessitates a dynamic re-weighting of sensory and motor cues. In early prosthetic users and older adults, decreased precision in proprioceptive signals or in the efference copy of the motor command may force the system to rely more heavily on external visual or intentional outcome cues to maintain a coherent sense of both agency and ownership (Jahanian Najafabadi et al., 2023b).

## The relation of agency and ownership

**Fig. 1:**
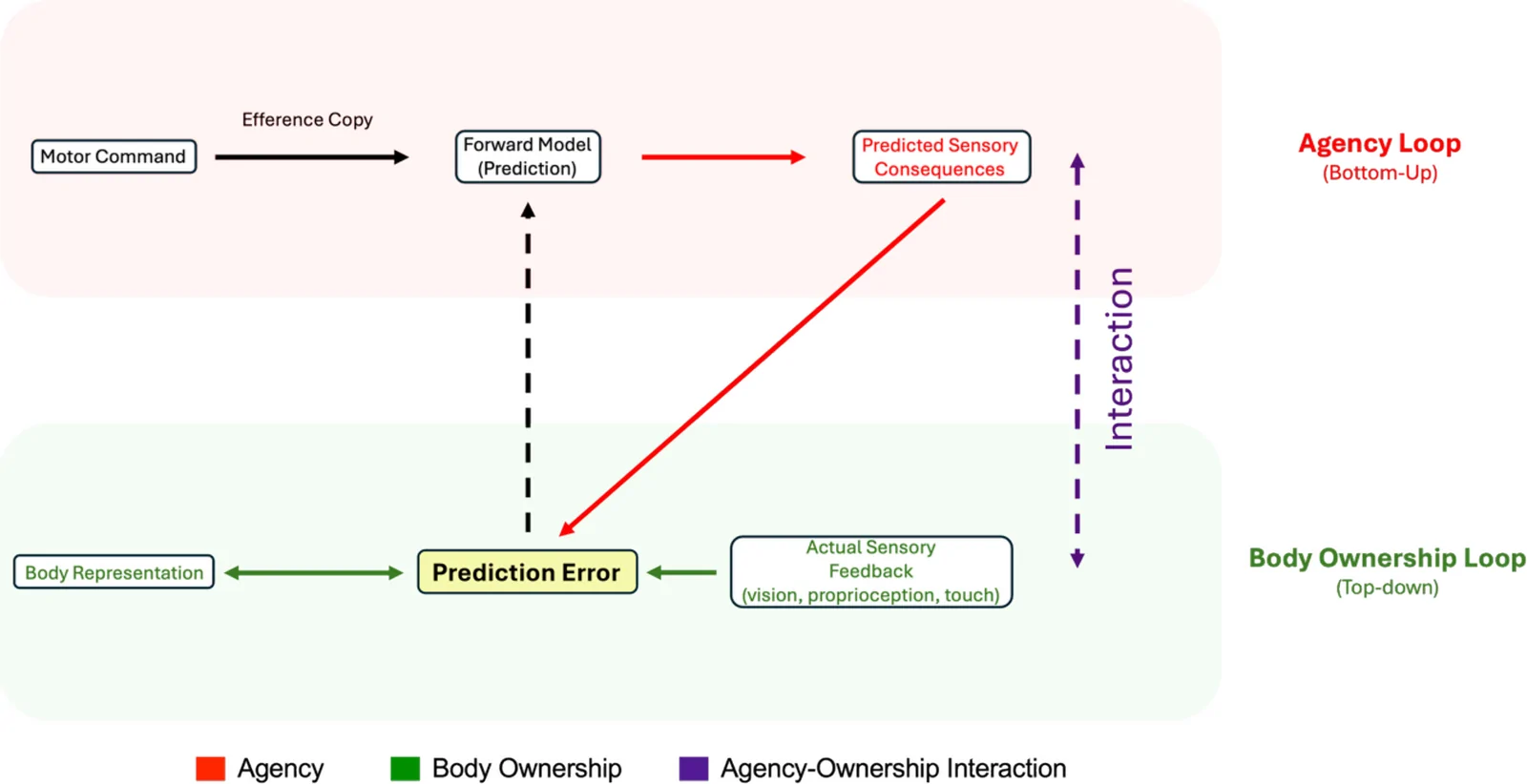
A motor command and the accompanying efference copy feed into the forward prediction model, producing the predicted sensory consequences. A comparator integrates the predicted sensory consequences and the actual sensory feedback from the action, giving rise to agency. Multisensory integration from different sensory feedbacks (vision, proprioception, touch), predictive information from internal body models (about the size and shape of the body), and action comparators contribute to body ownership. The figure highlights the Interaction (purple arrow) of the Agency loop (red) and Ownership loop (green). Agency emphasizes the prediction error minimization (did I cause this action?). Ownership emphasizes the integration with body representation (does this feedback belong to my body?). There is a bi-directional interaction between the agency and the ownership loop. Strong ownership reduces uncertainty in agency, and strong agency reinforces ownership

Agency and ownership can be conceptually unified under the predictive processing framework (Clark, 2013; Friston, 2010). From this perspective, both senses are products of the brain’s continuous effort to minimize the prediction error across different levels of the self-representation hierarchy (see Fig. 1). The sense of agency primarily minimizes the sensorimotor prediction error (i.e., did my action match my motor command?), while the sense of ownership minimizes bodily state prediction error (i.e., does the current multisensory input cohere with the expected body structure and state?) (Apps & Tsakiris, 2014). This shared computational goal helps to explain why these senses are often correlated and provides context for the specific neural networks involved.

Agency and ownership, though closely related in shaping the sense of self, arise from distinct neural and physiological processes. Agency originates in the neural mechanisms that govern motor actions, with the right frontoparietal network playing a pivotal role (Kang et al., 2015; Tsakiris et al., 2010). These networks are involved in planning, initiating, and monitoring actions, and thereby support the pre-reflective experience of being the author of one’s action. Agency thus hinges on motor control and the brain’s ability to predict and verify the outcomes of intentional actions, creating a seamless connection between intention and execution.

Ownership, on the other hand, is grounded in the sensory and interoceptive processes that allow an individual to perceive their body as their own. It emerges from afferent signals originating in internal bodily organs and the integration of interoceptive information, which conveys the state of the internal body. These signals are dynamically combined with multisensory inputs from peripheral sources, such as touch, proprioception, and vision, within the context of higher-order bodily representations (Makin et al., 2008). This multisensory processing supports the construction of a coherent sense of the bodily self, distinguishing one’s body from external objects or others. The interplay between agency and ownership highlights the complexity of self-perception, as these processes operate both independently and interactively. While agency ensures a sense of control over actions, ownership secures the recognition of the body as the entity through which those actions are executed. Collectively, these processes contribute to embodied self-awareness by maintaining a coherent representation of the body and supporting adaptive interactions with the environment.

However, the relationship between ownership and sense of agency are not fully solved, with ongoing debates about whether they are connected or distinct phenomena. Gallagher (2000) proposed that ownership and agency are two separated but closely linked components of the self. He emphasized that ownership arises from the integration of sensory signals from the body and the environment (Gallagher, 2005; Tsakiris et al., 2007). However, experimental studies have produced conflicting results on this topic (Kalckert & Ehrsson, 2014; Ma & Hommel, 2015a).

Ownership is thought to result from the integration of top-down and bottom-up factors, as outlined by Synofzik et al. (2008), an idea that was later explored by Ma and Hommel in a virtual reality context (Ma & Hommel, 2015a, 2015b; Ma et al., 2019). According to Synofzik et al. (2008), agency judgments can sometimes rely solely on bottom-up information, underscoring the importance of sensory inputs in shaping the experience of agency. Ma and Hommel (2015a) also demonstrated that non-corporeal objects could be perceived as parts of the body, suggesting flexibility in how external objects are matched with internal representations. Essentially, any object may be perceived as part of the body if it meets certain conditions, such as control over its behavior and a connection to the person’s body. In a follow-up study using the virtual hand illusion paradigm, Ma and Hommel (2015b) observed stronger skin conductance responses during synchronous conditions when participants controlled a virtual hand compared to a virtual rectangle. They found that illusory ownership increased with visuo-tactile stimulation and active control of a virtual effector, strengthening the tendency to perceive an object as part of the body. Although the study successfully induced rubber hand-like ownership illusions, it did not find evidence of objective agency during active manipulation of the virtual effector. However, they reported a significant correlation between subjective agency and perceived ownership, highlighting the complex interplay between these two aspects of self-representation.

Ma and colleagues (2019) further provided a critical theoretical extension to our understanding of the bodily self by applying Wegner’s agency theory to the virtual hand illusion. Their study demonstrated that both body ownership and agency are modulated by the principles of consistency and exclusivity, showing that participants experience stronger embodiment and control when the virtual effector’s movements align with their intentions (consistency) and when they perceive themselves as the sole cause of the action (exclusivity). Their findings reveal a significant interaction between consistency and exclusivity. While exclusivity enhances the illusion in the absence of motor consistency, the relevance of consistency becomes stronger when exclusivity is absent (Ma et al., 2019). When the virtual effector moves consistently with the participant’s own action, the participant already has strong evidence that “this is caused by me,” so exclusivity adds little. But when movement consistency is weak or absent, exclusivity becomes more important for agency and ownership judgments. This suggests that the brain utilizes a compensatory integration of multiple informational sources to maintain a coherent sense of self, providing a robust framework for interpreting how ownership and agency rely on overlapping sensory-motor cues.

Evidence that non-corporeal objects could be perceived as parts of the body not only in virtual reality but also in the real world, comes from a study applying the classical RHI setup to different objects with varying past agency experiences, like smartphones, a black block looking like a smartphone but with no past agency experience, a computer mouse, and a rubber hand (Liepelt et al., 2017). Synchronous and asynchronous temporal visuo-tactile stimulation was delivered to these objects, measuring the proprioceptive drift and ownership experiences. The study found that a rubber hand, a smartphone, and the black block could all elicit illusory body ownership, as indicated by higher ownership ratings following synchronous than asynchronous stroking. The proprioceptive drift, however, was sensitive to the past agency experience, showing that subjects perceived their unseen hand as being closer to the smartphone and the rubber hand, but not to the non-functional block, which people had no previous agency experience with. These findings suggest the role of past agency experience on the development of ownership over an object suggesting a certain level of interdependence between agency and ownership.

In this line, a later study by Lin et al. (2023) demonstrated that modern technological devices like smartphones can modify the body schema and extend body representation. Through a series of experiments utilizing stimulus–response compatibility and hand mental rotation paradigms, the study revealed that the brain integrates smartphones into its internal representation of the hand more effectively than traditional tools or non-smartphones. Furthermore, utilizing the forearm bisection task, the authors found that even the mental imagery of smartphone use leads to a perceived elongation of the forearm. This suggest that our functional “reach” for information and social connection provided by these devices is embodied as a physical extension of the self. Ultimately, these findings underscore that smartphone embodiment is a pervasive phenomenon in modern users, reaching a functional ceiling regardless of reported usage frequency.

That agency and body ownership are interconnected constructs is also supported by the central role of action effects in controlling intentional movements (James, 1890). The ideomotor model explains the learning process: intentional action is based on a Hebbian association between movement and its sensory feedback (Elsner & Hommel, 2001). This learning allows us to select actions by activating their desired sensory outcome (Hommel, 2015; Hommel et al., 2001), effectively constructing the necessary database for intentional action (Verschoor & Hommel, 2017). This ideomotor database provides the foundation for predictive coding models (Wolpert et al., 1998). In these models, the sense of agency is determined by comparing the predicted sensory consequences of an action with the actual sensory feedback (Frith et al., 2000b). Applying these same principles, the learned action effects derived from social and physical interactions also extend their impact to determine body ownership (Prinz, 2012; Verschoor & Hommel, 2017). The functional relationship between agency and ownership is clearly demonstrated in active RHI protocols, where self-produced movements are sufficient to induce the experience of owning an external limb (Tsakiris et al., 2005).

Kalckert and Ehrsson (2012) used a latex gloved wooden model of a hand whose finger movements were temporally synchronized or asynchronized with the movements of the subject’s occluded hand. These movements were either actively generated by the subject or passively driven by the experimenter. While synchronous movements triggered a strong sense of ownership over the model hand in both active and passive conditions, a strong sense of agency over its movements was only experienced when the subjects actively generated the movements. When the synchronous movements were initiated by the experimenter (passive movements), the sense of agency was abolished, while the sense of ownership remained intact. Conversely, when the hand model was rotated by 180 degrees, becoming anatomically implausible for the subject, the sense of agency remained, but the sense of ownership was eliminated, suggesting a dissociation of ownership and agency perception. Interestingly, these findings show that the sense of agency can be generated for an object that is not embodied.

Exactly how these results are generalizable is controversial and highlights the importance of considering context-specific embodiment mechanisms, especially when translating these concepts into applied domains such as prosthetic design or lifespan interventions. For example, Shibuya et al. (2017) showed that the interdependence of agency and ownership can vary with the task used. When a position-judgment task using a ruler or a visual-reaching task is used to measure proprioceptive drift, the amount of drift is modulated by agency and ownership. In a bilateral matching task, only ownership modulates the drift.

Taken together, these studies suggest that under certain conditions there is a modulation of ownership via agency and common information is used by both. Subject to the amount and type of available information, one should expect that some factors that influence the strength of agency should also influence ownership. Based on ideomotor theorizing, these factors are likely related to (a) *priority*, the proximity of cause and effect (Wegner, 2003); (b) *consistency,* the similarity between consequences and their cause; and (c) *exclusivity*, the absence of other possible causes of an effect (Wegner, 2003). Studies using RHI paradigms have been able to show that priority and consistency are constraints on ownership; however, the RHI setup is a very specific and unnatural situation (Costantini & Haggard, 2007; Kalckert & Ehrsson, 2014; Kilteni et al., 2015). The virtual reality version of the RHI, which allows free natural movement, could provide richer sources of information and better ecological validity. With the virtual reality variant of the RHI, Ma et al. (2019) provided evidence that just like for agency, exclusivity can also modulate ownership (Ma et al., 2019).

## Interconnections of body image, body schema, agency, and ownership

Throughout this review, we use *body ownership* to refer to the subjective experience of a body or body part as “mine”; *body schema* to denote action-oriented sensorimotor representations of the body; and *body image* to refer to more consciously accessible perceptual and conceptual body representations. We use *embodiment* as a broader term encompassing the integration of bodily or external effectors into the bodily self, which may involve ownership, agency, and changes in body representation. *Tool incorporation* refers more specifically to functional changes in body-related sensorimotor representations following tool use and does not necessarily imply subjective ownership of the tool.

Under the umbrella of the concept of minimal self, the concepts of a *sense of agency* and *ownership* of one’s experiences have proven valuable in connecting body representations to bodily consciousness (Arzy & Schacter, 2019). These phenomena provide foundational insights into how individuals perceive and interact with their physical form and environment. Moreover, the development of body representations is essential for the formation of the minimal self, including body ownership and agency (Georgie et al., 2019). In this context, body representations act as a bridge, linking physical self-perception to more abstract levels of consciousness that define human self-awareness. Moreover, the intricate relationship between body representations, body ownership, and agency underscores the dynamic nature of the minimal self. This self-awareness evolves continuously through interactions between the body, brain, and environment. These aspects are not only interconnected but also important for the cohesive experience of selfhood.

Based on the dyadic model, there are at least two representations of the body: *body image* and *body schema* (De Vignemont, 2010; Dijkerman & De Haan, 2007; Gallagher, 1986). As outlined, both concepts, body image and body schema, were proposed to be two conceptually distinct concepts by Shaun Gallagher in the 1980s (Gallagher, 1986). The complexity of body representations has been a topic of discussion since the early twentieth century, with efforts made to clarify the concept of self-representation and its varying interpretations (De Vignemont, 2010; Gallagher, 1986).

Body image refers to a conscious, perceptual representation of the body, including sensory details such as size and shape. It is associated with how we perceive and emotionally relate to our bodies (Cardinali et al., 2012). Body image also encompasses long-term aspects of self-representation, serving as a stable framework for body perception, body affect, and body concept over time, and represents a long-term conscious representation of the body which is required for action initiation (Ataria et al., 2021; Cardinali et al., 2012; Gallagher, 2005). In contrast, body schema represents an unconscious, sensorimotor framework that enables action planning and movement execution (D’Angelo et al., 2018; Martel et al., 2016). Body schema operates as a short-term, dynamic representation that guides bodily movements and contributes to the sense of ownership by providing the spatial content of bodily sensations (Cardinali et al., 2012; Jahanian Najafabadi et al., 2021, 2026a). Its inherently plastic nature allows body schema to adapt continuously, evolving alongside body image throughout an individual’s lifespan (D’Angelo et al., 2018). Together, body image and body schema form the broader concept of body representations and serve as a cognitive framework for understanding self-representation. However, despite this distinction, these terms still overlap, making them challenging to separate clearly in some contexts.

Following Gallagher, new models have been proposed relating this distinction to explicit and implicit levels of bodily processing (De Vignemont, 2010; Longo & Haggard, 2010; Salomon, 2017; Tsakiris et al., 2010). For example, Longo and Haggard (2010) proposed that these two types of body representations can be dissociated theoretically and empirically. The *body image* refers to our conscious, subjective experience of the physical form and appearance of our body. Complementary body representations depict the body in a spatially ordered manner (Medina & Coslett, 2016). The body image is relatively stable across time and clearly accessible to conscious awareness. In contrast, the *body schema* is action-oriented, referring to a more dynamic representation of our body posture and orientation (Holmes & Spence, 2004), and is less accessible to conscious awareness and introspection (Longo & Haggard, 2010).

A recent study by Erkent et al. (2025) focused on the dynamic interaction of body image and body schema, demonstrating that visual manipulation of tool length can modulate motor representation of forearm size, a theoretical perspective extensively investigated by prior research attempting to address motor and perceptual embodiment using tool-use paradigms. In their study, Erkent et al. (2025) tested whether perceptual modifications to body representation versus movement can induce motor changes or vice versa, in which participants were required to perform active tool use of varying size (of short and long tools) to move objects versus passively holding a tool while watching a rubber hand holding a matching tool. Using a rubber tool illusion paradigm, when the stroking on both tools’ tips was synchronized, participants holding the short tool perceived their own forearm as longer if the rubber hand used a long tool. Control tests showed this effect only occurred if people had previously used the tools actively, felt ownership over the rubber hand/tool, and observed a length mismatch between what they held and what the rubber hand held. These findings support the hypothesis proposed by Cardinali et al. (2009) and Martel et al. (2016) in which perceptual body image and body schema are intercorrelated and influence each other.

Previous studies (see Limanowski & Blankenburg, 2013; Limanowski, 2014) have primarily focused on inducing short-term plastic changes in body representations through body ownership illusions while exploring the neural relationships between agency and ownership. Under certain conditions, the sensation of owning a body can led to self-attribution of actions, such as speaking (Banakou & Slater, 2014).

Research utilizing functional brain imaging in body ownership illusion studies highlighted the significance of integrating multisensory information (e.g., visual, tactile, proprioceptive, and motor inputs) in the experience of agency and ownership (e.g., Tsakiris & Haggard, 2005). These studies have identified key neural regions involved, including the premotor cortex, supplementary motor area, somatosensory cortex, intraparietal sulcus, and posterior parietal cortex (e.g., Baier & Karnath, 2007; Blanke et al., 2002; Tsakiris et al., 2008). Additionally, brain areas like the anterior and posterior insula, anterior cingulate cortex, and cerebellum are implicated in illusory ownership (Evans & Blanke, 2013). Specifically, the right posterior insula has been linked to the experience of body ownership (Baier & Karnath, 2007).

Efforts to clarify the neural basis of body representations have also examined patient populations (Schwoebel & Coslett, 2005). For example, an experiment using the RHI revealed that stroke patients displayed stronger ownership and agency experiences compared to healthy controls, despite hemiparesis and sensorimotor cortical reorganization. This suggests that, in these patients, visual input may take precedence over proprioceptive signals (Llorens et al., 2017). Similarly, studies have found that hemiplegic patients exhibit greater flexibility in ownership for the affected hand, whereas the healthy hand shows more rigid ownership, indicating that sensorimotor experiences and frequency of use can influence body schema strength (Burin et al., 2015).

More recent studies have employed electrophysiological techniques to examine brain activity and connectivity during body ownership illusion tasks. For instance, suppressed beta power in the sensorimotor cortex and enhanced beta connectivity between the sensorimotor cortex and inferior parietal cortex were observed during illusory hand ownership experiences (Faivre et al., 2017). Additionally, suppression of beta and alpha power in frontal areas and superior regions of the supplementary motor area has been reported in similar tasks (Rao & Kayser, 2017). These findings suggest that higher-order brain regions, rather than just sensorimotor areas, play a role in mediating illusory ownership, particularly the intraparietal and premotor regions.

While fine motor skills, including tool use, tend to decline with age (Diermayr et al., 2011; Jahanian Najafabadi et al., 2023b, 2026a), older adults can still improve their motor performance with practice (Jahanian Najafabadi et al., 2023b, 2025; Lazarus & Haynes, 1997; Lutz et al., 2010; Seidler, 2007; Voelcker-Rehage & Alberts, 2005). In some cases, older adults even show stronger learning effects compared to younger adults (Bock & Schneider, 2001, 2002). This highlights the critical role of practice in acquiring and refining new skills (Studer et al., 2010), particularly for older individuals.

Motor learning is broadly defined as any improvement in performance driven by experience (Krakauer et al., 2019). It involves achieving greater spatial and temporal accuracy in movements through practice, leading to skill acquisition. Examples of motor learning include everyday tasks such as using a knife to cut fruit, using a rake to reach an object, or manipulating tools in the environment. Beyond these day-to-day tasks, tool use also extends to specialized contexts like surgery, where mastering the skill often requires years of training (Maravita & Romano, 2018).

## Psychophysical approaches to characterising embodiment across the lifespan

Because the precision and relative weighting of sensory information change across development and aging, psychophysical approaches provide an important means of characterizing lifespan differences in the bodily self. While traditional investigations into the bodily self have relied heavily on subjective questionnaires and proprioceptive drift, a significant shift in contemporary literature involves the application of formal psychophysical models to characterize embodiment. These approaches are particularly valuable as they isolate genuine shifts in perceptual sensitivity from variations in response bias or decision criteria. This move toward more quantitative and rigorous methods addresses long-standing concerns regarding the susceptibility of traditional measures to demand characteristics, suggestibility, and response bias. Key to this advancement is the elaboration of a signal detection theory framework for body ownership, which allows for the independent measurement of a participant’s sensitivity to illusory sensations (their ability to discriminate ownership) versus their internal decision criterion or bias (Chancel & Ehrsson, 2020). By employing two-alternative forced-choice (2AFC) tasks, researchers have demonstrated that the temporal and spatial constraints of multisensory integration are significantly tighter than previously believed. This suggests that ownership discrimination is a highly sensitive process governed by precise multisensory alignment (Chancel & Ehrsson, 2020). Chancel and Ehrsson proposed that body ownership is a true perceptual multisensory experience that can be measured using psychophysical methods in discrimination studies. It is intricately linked to the brain’s capacity to integrate sensory, motor, and cognitive information, providing a cohesive and embodied sense of self.

Building upon this, recent computational modelling has framed body ownership as a process of causal inference, where the brain must decide whether visual, tactile, and proprioceptive signals share a common cause (the self) or originate from different sources. Chancel et al. (2022) demonstrated that the emergence of the RHI is well predicted by an uncertainty-based inference model; specifically, as visual noise increases, the brain relies more heavily on prior expectations of a “common cause” increasing the probability of the illusion. This suggests that the subjective experience of ownership is the result of a Bayesian-like integration of sensory evidence and prior bodily representations, where the prior for ownership is stronger than the prior for simple multisensory synchrony.

Furthermore, Lanfranco et al., (2023, 2024) have extended these psychophysical approaches to demonstrate how specific sensory parameters, such as texture congruence, modulate perceptual bias without necessarily altering the underlying sensitivity to visuotactile stimulation. By fitting psychometric functions to RHI data, these studies provide a parametric quantification of the “point of subjective equality” (the threshold at which a hand is perceived as one’s own), offering an objective benchmark for how external objects are incorporated into mental body representations. Most recently, this framework has been utilized to dissociate the mechanisms of multisensory integration from conscious access itself. Lanfranco et al. (2025) utilized signal-detection analysis and drift–diffusion modelling to show that while the integration of signals is a prerequisite for ownership, the subjective experience of “mine-ness” involves additional evidence-accumulation processes. These methodological advances not only provide robust alternatives to traditional measures but also clarify the hierarchical relationship between low-level sensory processing and the high-level conscious experience of a unified bodily self. Importantly, this distinction is particularly relevant from a lifespan perspective. Developmental and age-related differences in embodiment may reflect changes in perceptual sensitivity, shifts in decision criteria, altered weighting of sensory evidence, or changes in prior expectations. Psychophysical and computational approaches provide a means of distinguishing these possibilities and therefore offer a particularly useful framework for determining how the mechanisms underlying agency, ownership, and body representation change across the lifespan.

## Pathological embodiment related to ownership and agency

A deeper understanding of ownership fundamentally shapes current perspectives in both neuroscience and psychology. However, to fully appreciate the underlying complexity of sensorimotor awareness, one must also examine clinical populations where the distinction between self and other is disturbed. While historically viewed as monolithic deficits, neurological conditions such as asomatognosia and somatoparaphrenia reveal a nuanced dissociation within the body schema.

Asomatognosia refers to a patient’s feeling that parts of his or her body are absent or have vanished from corporeal awareness. This phenomenological deficit is linked to the premotor cortex and the posterior parietal cortex (Arzy et al., 2006), particularly the temporoparietal junction (Feinberg et al., 2010). Somatoparaphrenia is a delusional belief of disownership in which contralesional body parts are experienced as not belonging to oneself, most commonly affecting the left side of the body following right-hemispheric lesions of the parieto-frontal-insular network (Gandola et al., 2012; Ronchi et al., 2018; Martinaud et al., 2016; Vallar & Ronchi, 2009). When the insular-parietal network is compromised, the brain fails to synthesize multisensory signals into an integrated internal body model, resulting in the “disownership” of the affected limb. There are also other cases of pathological embodiment leading to an inverse error in self-attribution. In these cases, patients do not deny their own limbs but instead “incorporate” an external hand, often that of a clinician, into their own body representation (alien hand). Neurologically, this condition is associated with damage involving the right premotor cortex and the frontal operculum, regions responsible for maintaining the inhibitory boundaries between the self and the environment (Garbarini et al., 2020). Notably, this “mis-ownership” is not merely a cognitive error, it is biologically anchored. Evidence from the hand-blink reflex demonstrates that pathological embodiment patients exhibit subcortical defensive responses to threats approaching an embodied alien hand, suggesting that these delusions are deeply rooted in the brain’s primal survival circuits (Fossataro et al., 2019).

A unique window into the dissociation of ownership and agency is provided by the anarchic hand syndrome (Della Sala et al., 1991). Unlike the dis-ownership seen in somatoparaphrenia, patients diagnosed with anarchic hand syndrome maintain an intact sense of ownership (“this is my hand”) but suffer a catastrophic loss of volitional control (Della Sala et al., 1991; Garbarini et al., 2015; Schaefer et al., 2010).

The frontal variant of anarchic hand syndrome, resulting from damage to the supplementary motor area and anterior cingulate cortex, manifests as compulsive, goal-directed actions that occur without conscious intention (Manea et al., 2024). This underscores a critical functional divide: while the parietal-insular network monitors whose body it is (ownership), the frontal-cingulate circuit remains the arbiter of who is initiating the movement (agency). Collectively, these pathologies indicate that the “sense of self” is a fragile, emergent property of a widely distributed network, where even minor disruptions in fronto-parietal or subcortical connectivity can lead to a radical restructuring of the bodily experience.

## Neurodegenerative disorders altering body ownership

This neural dissociation is further illuminated by recent evidence suggesting that the assimilation of external tools and biological limbs into a cohesive body schema is a hierarchical process, where ownership and agency are supported by divergent neural substrates. Traditionally, this integration was viewed as a predominantly cortical phenomenon; however, findings now emphasize the critical contribution of subcortical architectures, specifically the basal ganglia (putamen) and the cerebellum, in maintaining the stability of body ownership (Crucianelli et al., 2024). In neurodegenerative contexts like Parkinson’s disease, subcortical disruptions appear to “blur” the temporal constraints of multisensory integration. This results in a heightened susceptibility to ownership illusions, where patients may attribute ownership to external stimuli even during asynchronous sensory feedback (Waldmann et al., 2020). Notably, this instability in ownership does not necessarily mirror age-related declines in multisensory recalibration; rather, recent data indicate that aging, not Parkinson’s disease, may be the factor that decreases the recalibration of body ownership through vision-respiratory interactions (Shoji et al., 2024).

Instead, Parkinson’s disease-specific deficits are more closely tethered to an altered awareness of action, or sense of agency. Using both explicit action recognition tasks and implicit intentional binding paradigms, researchers have found that patients diagnosed with Parkinson’s disease show a reduced ability to integrate motor predictions with sensory feedback, failing to show the “intentional binding” effect (the temporal attraction between actions and their effects) seen in healthy controls (Saito et al., 2017). Ultimately, these data indicate that a functional sense of agency may be both neurobiologically dissociable from, and more instrumental for, motor precision than the subjective feeling of limb ownership. Specifically, an explicit sense of agency over a visible limb has been shown to improve the initiation of movement and eye-hand coordination, regardless of whether a sense of body ownership is present (Matsumiya, 2021). Such findings underscore the clinical imperative to develop rehabilitation protocols that move beyond gross motor training to target the nuanced proprioceptive and body awareness deficits inherent in neurological pathology (Cardile et al., 2025).

Beyond the structural disruptions seen in clinical pathology, the healthy body schema remains a remarkably flexible construct. Research indicates that the minimal self is likely to remain plastic in a mature human brain, which is based on normal early cognitive development (Hommel & Kibele, 2016; Yee & Bailenson, 2007). Hence, it is acceptable to expect that everyday activities that engage short- and long-term motor activity and sensory input can shape our minimal self. Due to age-related changes in the brain throughout life, it is essential to explore how brain functions differ across various age groups.

## The early development of bodily selfhood

The development of the bodily self is a protracted process characterized by a high degree of early multisensory flexibility. This process begins as early as the first days of life, as evidenced by newborns’ ability to detect visual-tactile contingencies. Infants as young as 12 to 103 h old exhibit a preference for synchronous multisensory information, suggesting that the basic mechanisms for body perception are present early from birth on (Filippetti et al., 2013). This early sensitivity forms the foundation for differentiating the self from the external environment long before significant postnatal experience has occurred (Bremner et al., 2012).

As children grow, they exhibit greater plasticity in integrating internal body signals with external sensory information compared to adults, as they must constantly recalibrate their body schema to accommodate rapid physical growth (Cowie et al., 2013). During middle childhood (ages 4–9 years), children show a significantly higher susceptibility to bodily illusions, such as the RHI, because they rely more heavily on visual capture and have a less stable “internal” proprioceptive map than adults (Cowie et al., 2013). This is further supported by evidence that preterm birth may specifically impact this developmental flexibility; preterm children often report lower scores of subjective embodiments over illusory stimuli, suggesting that early sensory experiences in critical periods significantly influence the neurobiological mechanisms underpinning the sense of one’s own body (Della Longa et al., 2021).

The underlying integration processes are notably different from the “statistically optimal” integration seen in adults. Research indicates that prior to 8 years of age, children exhibit suboptimal integration of visual and haptic spatial information. Instead, one sense typically dominates. For example, haptic information dominates for size discrimination, while vision dominates for orientation, regardless of which sense is more precise (Gori et al., 2008). This lack of optimal integration in young children is actually considered a developmental advantage; it allows the brain to resolve sensory mismatches while the body’s physical dimensions are in flux by using the dominant sense to “train” the others (Gori et al., 2008).

Furthermore, research using the RHI with children has identified dissociable pathways in body representation. While 4-year-old children are already as sensitive as adults to visual-tactile synchrony cues indicating an early-maturing pathway for explicit embodiment, they demonstrate a later-maturing process for visual-proprioceptive integration (Cowie et al., 2013). Regardless of synchrony, children’s perceived hand position is typically closer to the rubber hand than that of adults, reflecting a more malleable and vision-dominated spatial representation of the self in children (Cowie et al., 2013). This heightened adaptability is crucial for the successful development of self-awareness, ensuring that the bodily self remains cohesive despite the physiological and sensory changes inherent in early development.

In contrast, adults rely more on well-developed cognitive strategies to manage these discrepancies, influencing their sense of body ownership and motor skill development (Liesner et al., 2021). This fundamental difference in sensory processing between children and adults plays a key role in shaping body representation and self-awareness over time. Moreover, Martel et al. (2021) suggested that adolescents exhibit distinct patterns of body representation updates compared to adults. These differences may stem from immature motor control, underdeveloped multisensory integration, and rapid physical growth before and during puberty. The typical kinematic patterns seen in adults, often associated with tool incorporation, appear to be delayed in adolescents due to their slower developmental trajectory. The pubertal growth spurt disrupts the steady development in childhood, leading to reduced kinesthetic precision and motor proficiency during early puberty (Assaiante et al., 2014; Roy et al., 2013). This rapid growth also impacts proprioceptive integration, which matures gradually over time (Cignetti et al., 2013; Viel et al., 2009). Unlike adults, adolescents rely more heavily on visual information and less on proprioceptive cues, a critical factor for posture control and tool use (Assaiante et al., 2014; Cardinali et al., 2016; Martel et al., 2019). In contrast, older adults with heightened interoceptive sensitivity tend to exhibit reduced plasticity in body schema. This suggests that greater awareness of internal bodily sensations may correlate with decreased body schema plasticity as age increases (Raimo et al., 2021), which is also highlighted by the findings reported by Jahanian Najafabadi et al. (2023b).

## The interplay of agency and ownership in healthy aging

**Fig. 2:**
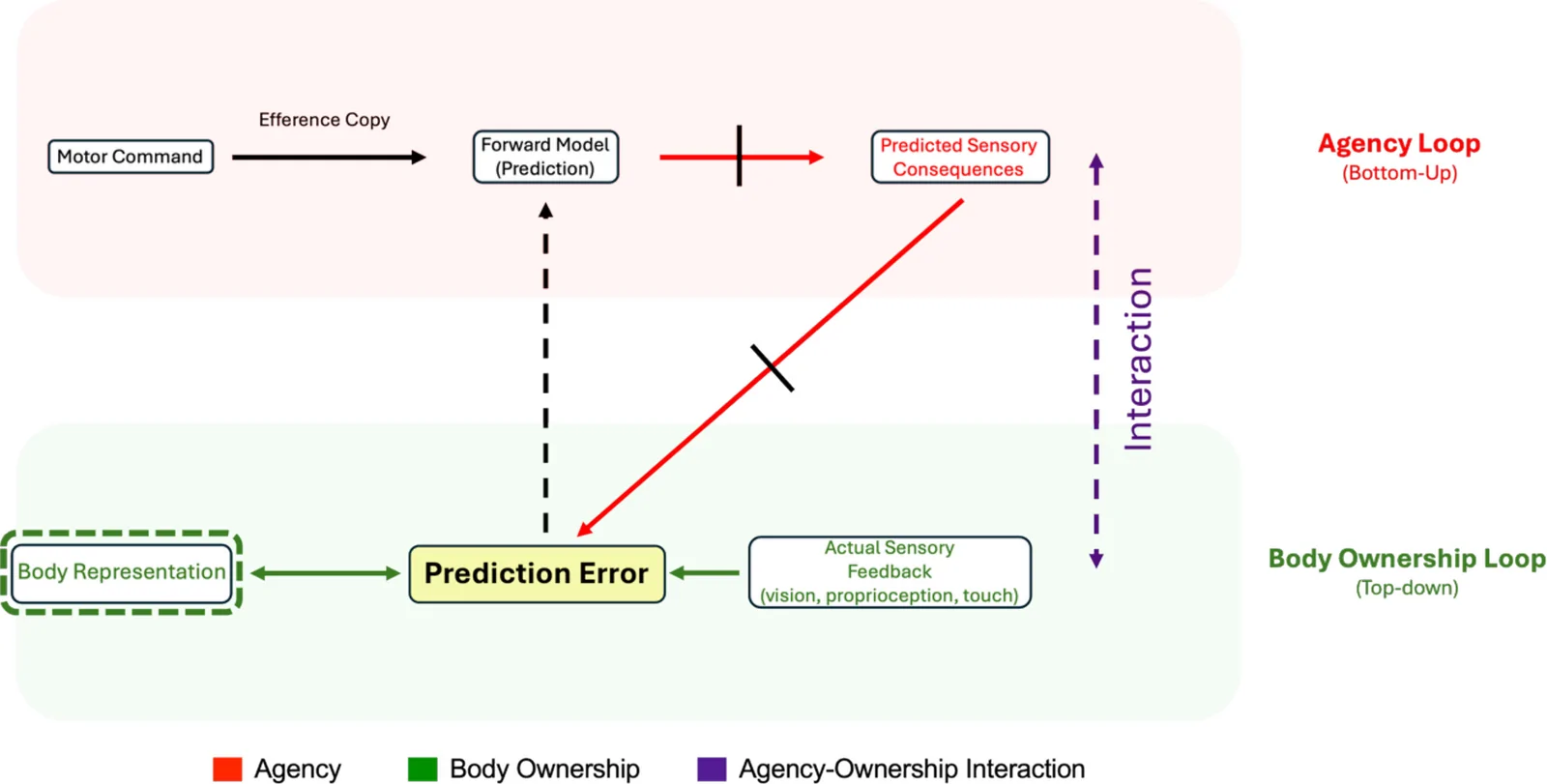
The figure shows the forward prediction model for aging adults. The forward prediction model may cause problems in predicted sensory consequences coming with age. Based on the comparator that integrates predicted sensory consequences with the actual sensory feedback from the action incorrectly, a larger prediction error arises. This may force the system to rely more heavily on external visual, proprioceptive, and touch signals or intentional outcome cues to maintain a coherent sense of both agency and ownership. In addition, internal signals related to body representation become less reliable with age. Compensation strategies may involve less weighting on internal bodily signals and more weighting on the actual sensory feedback. The assumption of a bi-directional interaction between the agency and the ownership loop may allow ways to free up cognitive capacity in aging individuals

As we age, the sense of our own body often changes. This can include less precise awareness of limb position (Han et al., 2016), difficulties combining information from different sensory modalities (Völter et al., 2021), and challenges with predicting and controlling movements and postural control linked to the sensorimotor integrative function (e.g., Henry & Baudry, 2019; Wang et al., 2024). Such impairments may lead to a feeling of being less connected to the body, trouble using tools effectively, difficulty adjusting to things like assistive devices, or difficulties in planning upper-limb movements to somatosensory targets as such motor planning processes in older adults rely on somatosensory cues from the effector limb (Goodman & Tremblay, 2021). By examining the developmental trajectory and interaction of agency and ownership across the lifespan, we can begin to understand and potentially address some of these age-related difficulties. In older adults, this predictive mechanism shows a significant decline (see Fig. 2). For example, in Mariano et al. (2024), older adults exhibited a reduced explicit and implicit sense of agency, suggesting that older adults, compared to persons of younger age, are more reliant on internal predictions by counting more on precise and accurate interoceptive and proprioceptive information, which in turn leads to reduced cognitive biases and external manipulation.

Recent research by Jahanian Najafabadi et al. (2026a) challenges the prevailing assumption that brief tool use naturally triggers body representational plasticity. Across different age groups, participants consistently underestimated tactile distances on the forearm; however, short-term tool use training failed to produce significant shifts in these tactile judgments or in perceived reaching distances. These results suggest that while the literature often highlights tool-driven adaptations, brief exposure may be insufficient to induce measurable sensorimotor changes. Consequently, more prolonged or immersive training may be required to alter body representation, which appears to remain fundamentally stable across the lifespan despite age-related improvements in tool proficiency. In a subsequent study, Jahanian Najafabadi et al. (2026b) found that adolescents and young adults report a significantly higher subjective sense of tool ownership than other age groups, independent of tool size or training distance. Notably, in these younger populations, higher ownership ratings over the tool were predicted by a reduction in forearm tactile distance estimation errors, particularly after training in far distance, compared to middle-aged adults. While the sense of agency was predicted by perceived reaching distance across all participants, the feeling of ownership over the tool was uniquely tied to the interaction between reaching distance and tactile perception. Collectively, these findings suggest that sensorimotor-based embodiment is jointly shaped by tactile and spatial perception, with these effects peaking during adolescence and young adulthood.

Further studies exploring how the sense of agency and body ownership evolve and interact across the lifespan shed light on these age-related challenges. For instance, Zapparoli et al. (2022) found that older adults tend to experience a reduced sense of agency, suggesting that our ability to feel in control of our actions develops gradually, peaks in adulthood, and then declines in later life. This pattern resembles the inverted U-shaped curve often seen in other aspects of motor function across the lifespan. A later finding (Mariano et al., 2024) revealed that older adults displayed a diminished explicit and implicit sense of agency, suggesting a greater reliance on internal predictions and more precise interoceptive and proprioceptive signals. This shift may help to reduce cognitive biases and make older adults less susceptible to external manipulations.

Similarly, Cioffi et al. (2017) showed that older adults experience a reduced sense of vicarious agency. This finding was interpreted to be linked to a greater, and perhaps compensatory, dependence on internal, self-specific cues to define agency. This shift in cue reliance provides insights into how the sense of agency changes with age. These results support the broader notion that the neurocognitive processes driving the sense of agency shift with age, leading to a decreased sensitivity to environmental manipulations (e.g., temporal or spatial discordance between action and effect), which manifests as a reduced prediction error signal. These results further support how older adults experience a reduced sense of agency with age. Even when delays or distortions were added, their sense of control stayed mostly the same, indicating changes in older adults’ metacognition of agency compared to the younger ages (Metcalfe et al., 2010). This relative insensitivity suggests an age-related degradation of the internal forward model, leading to a diminished ability to effectively integrate predicted consequences with actual sensory feedback.

These age-related changes in the sense of agency are closely intertwined with the sense of body ownership as the feeling that one’s body and its movements truly belong to oneself (Cioffi et al., 2017; Hidaka et al., 2021; Marotta et al., 2018). Overall, these studies using the RHI have shown that older adults may experience a weaker sense of body ownership compared to younger individuals, and that the internal configuration of ownership and agency sensations across different body parts becomes less distinct with age. This suggests that the processes underlying agency and ownership are interconnected and both are subject to age-related alterations.

While prior research links the sense of ownership over the body or environment to the integration of multisensory inputs, older adults generally demonstrate a preserved capacity for corporeal awareness. For example, the RHI can still be successfully induced in healthy older adults (Ferracci & Brancucci, 2019; Marotta et al., 2018). However, the mechanism supporting a sense of ownership is less precise: the temporal binding window defined as a period within which different sensory stimuli must occur to be perceived as simultaneous is often widened in older adults (Chan et al., 2015). This broadened temporal window may reflect an age-related reduction in the temporal precision of neural processing or a compensatory strategy to group ambiguous signals.

Body schema and body image, i.e., visuo-spatial body map, were found to be related to conscious body ownership and agency, i.e., the conviction to control one’s own body and all its parts (Cardinali et al., 2012; De Vignemont, 2007). These sub-components play different roles: While the body image is crucial during the initial phase of motor learning, the body schema serves as a necessary prerequisite for advanced motor learning phases, enabling the implicit adaptation of novel motor patterns updating constantly (Ataria et al., 2021; Tanaka, 2021). Previous studies revealed the plasticity of body schema with movements of body parts, the emergence of ownership and agency over the body parts (for review, see Braun et al., 2018; Kong et al., 2017; Rubo & Gamer, 2019) as well as the development of agency over the virtual limb (D’Angelo et al., 2018).

With age, changes in perception, movement, and cognition become critical (Allen & Roberts, 2016; Dexter & Ossmy, 2023). Some of these changes stem from declines in both central and peripheral nervous system functions, while others are shaped by shifts in how individuals engage in sensorimotor activities, use their cognitive and physical resources, or interact with their surroundings (Lee & Kim, 2022; Yoshimura et al., 2020; Zimmerman et al., 2021). Age-related development also varies significantly from person to person, depending on their personal experiences and capacities. However, many aspects of these changes coming with age remain adaptable and can improve through targeted training or stimulation (Lustig et al., 2009; Sánchez-Cazalla & Gutiérrez-Domingo, 2025). Together, these factors influence how older adults interact with their environment and how they perceive information from both their bodies and the outside world. This makes older adults a particularly valuable group for studying the adaptability of the minimal self, offering opportunities to explore how age-related neural and behavioral changes interact with training, development, and life experiences.

Due to age-related sensorimotor decline and the changes in sensory perception, the perception of the minimal self can also be influenced. For example, prior research (for review, see Kuehn et al., 2018) has reported that the degradation of internal models in older adults may lead to impairment in cognitive and social abilities, which is also associated with motor control and bodily attention. Such age-related impairments have been systematically linked to deficits in embodiment, a phenomenon underpinned by progressive neuronal degradation within sensorimotor regions (Kuehn et al., 2018).

The functional impact of these age-related declines is acutely tested during tool use, a process that fundamentally requires the extension and transient modification of the body schema to incorporate the tool (Maravita & Iriki, 2004). This requires the sensorimotor system to create an internal tool model. Studies investigating tool use in aging provide mixed evidence: While prior research (e.g., Wolpe et al., 2020) demonstrated that older adults can still show significant motor adaptation and retention when learning to use novel tools or distorted feedback, suggesting preservation of cerebellar and cortical plasticity, the efficiency of this process is often reduced. Jahanian Najafabadi et al., (2023a, 2023b) showed that older adults exhibit less robust changes in the body schema following tool use compared to younger individuals. Specifically, the neural representation of the tool’s length or affordance, which typically rapidly integrates into the somatosensory cortex in younger adults, appears to be less stable or requires more extensive training in older adults.

Specifically addressing fine motor control in complex actions, Hooyman et al. (2021) demonstrated that the general slowing of tool use in older adults is driven primarily by prolonged duration and reduced quality in the fine motor phase (object acquisition), with the gross motor phase remaining relatively intact. Older adults exhibited less efficient fine motor control, characterized by greater spatial variability (increased ellipsoid volume of tool-motion), which was directly correlated with longer movement times. Crucially, this decline in fine motor efficiency was linked to lower cognitive scores in the older group, reinforcing those age-related changes in complex movement, which depend on an integrated sense of agency and an adapted body representation, are associated with cognitive status.

To understand the mechanics of this decline, for example, one must consider the neurocognitive principles of consistency and exclusivity that govern the sense of self. Research indicates that body ownership and agency are modulated by how well an external effector’s movements match one’s intentions (consistency) and the absence of other salient causes for that action (exclusivity). In older adults, the increased spatial variability in fine motor control acts as internal “noise,” disrupting the visuomotor consistency required for stable embodiment. As reported (Ma et al., 2019), consistency can compensate for a lack of exclusivity; therefore, providing high-consistency feedback may be a vital strategy to override internal model degradation in the elderly. The plasticity of the body schema remains a powerful tool across the lifespan, as evidenced by the phenomenon of smartphone embodiment. Studies showed that modern tools can be integrated into the body representation (Liepelt et al., 2017) to the extent that they physically extend the perceived length of body parts, such as the forearm (Jahanian Najafabadi et al., 2023a; Lin et al., 2023). This suggests that the brain’s body representation is flexible enough to incorporate tools based on functional affordance and the sense of “connection” they provide. However, successful embodiment requires integrating visual and proprioceptive information in an overlearned manner, a process that may become less stable in older adults.

Ultimately, these findings suggest that interventions, such as optimizing assistive technologies, must focus on supporting the precision and efficiency of fine motor control to mitigate functional decline. By prioritizing technologies that offer intuitive, high-consistency motor feedback, we can ensure that the body’s predictive control mechanisms can successfully integrate and maintain a coherent, efficient body representation across the lifespan.

## Future directions and implications

The studies outlined in this review have several implications for our understanding of agency and ownership and associated bodily components. The first implication is that multisensory integration and the construction of the minimal bodily self has a bottom-up component related to perceptual information coming from visual, proprioceptive, and tactile modalities being sensitive to the relative temporal stimulation. The second implication is that the self is sensitive to differences between the internal body model and these incoming signals. The third implication is that the perception of agency and ownership might be linked together, and that this linkage seems to depend on the amount and quality of available information at hand. However, as this review has highlighted, this interdependence is yet to be thoroughly investigated.

These implications are, however, based on experiments using quite different methods and paradigms which are heterogenous. The way in which body ownership is measured may have produced different outcomes across studies assuming that these measurements do not always measure the same underlying construct but may reflect different aspects of the illusion. The aforementioned difficulties in studying the self are likely to surface in more applied research, such as creating prosthetic and bionic limbs. Understanding how agency experiences affect ownership and multisensory integration could optimize the integration of prostheses or exoskeletons into our bodily self. Prostheses that respond consistently to the user’s motor intentions may facilitate both agency and their functional incorporation into body representations, which could help to minimize the prediction error (see Fig. 2). Over time, these processes may contribute to a broader experience of prosthetic embodiment. Conversely, the optimization of these bodily experiences via body schema plasticity represents a compelling avenue of future research, as a growing body of literature investigates these dynamic updates. The linkage between agency, ownership, and action control could also provide new ways to remotely control and interact with robots and machines more directly with our bodies and our minds through brain-machine interfaces. These technologies have many potential uses ranging from remote surgeries to space control.

Additionally, researchers must develop and test models that quantify the relative precision (reliability) assigned to sensory, motor, and cognitive cues across the lifespan. This would involve using Bayesian or reinforcement learning models to predict how much a change in proprioceptive precision (as seen in aging) or external haptic feedback (as seen in advanced prosthetics) should mathematically alter the strength of agency and ownership. Such modelling would enable the design of technologies that are not merely realistic, but computationally optimal for user integration.

A further critical area for future study involves mitigating the age-related decline in fine motor control and its associated cognitive load. Given that older adults show reduced fine motor efficiency linked to lower cognitive scores, future research should investigate whether enhancing agency and ownership cues can serve as a cognitive compensatory mechanism. For instance, optimizing the visual and haptic feedback of an assistive tool to maximize the sense of embodiment may lower the attentional resources required to control it, thus freeing up cognitive capacity. Longitudinal studies should examine interventions focused on strengthening body-tool interactions. For example, virtual reality training can sustain or improve functional independence and complex motor skills in older adults over time.

## Supplementary Information

Below is the link to the electronic supplementary material.

Supplementary file1 (DOCX 15 kb)

Supplementary file2 (DOCX 26 kb)

## Data Availability

Not applicable.
