# Supplementary material for: The dynamic bodily self across lifespan: Agency, ownership, and the plasticity of internal body models
Source: Psychon Bull Rev. 2026 Oct 9;33(8):254. doi: 10.3758/s13423-026-03024-0 (PMC13652922; doi:10.3758/s13423-026-03024-0)
Supplement: Supplementary file 1 [file 13423_2026_3024_MOESM1_ESM.docx]

**Zusammenfassung**

Zwei grundlegende Dimensionen der Forschung zur körperlichen Selbst-Bewusstheit, Agency (Handlungsurheberschaft) und Body Ownership (Gefühl des Körperbesitzes), haben in den vergangenen Jahren ein exponentielles Wachstum erfahren. Die wegweisende Arbeit von Botvinick und Cohen aus dem Jahr 1998 lieferte mit der Gummihand-Illusion, bei der man das Gefühl hat, ein externes Objekt werde zum Teil des eigenen Körpers, ein zentrales psychophysisches Instrument zur Untersuchung des körperlichen Selbst. Aktuelle motorische Kontrollmodelle der Ownership betonen den Einfluss von Agency, also dem Gefühl, die Kontrolle über die eigenen Bewegungen zu haben. Technologische Fortschritte haben ökologisch validere experimentelle Paradigmen wie virtuelle Versionen der Gummihand-Illusion hervorgebracht und damit unser Verständnis der Mechanismen vertieft, durch die das Gehirn Agency- und Ownership-Empfindungen erzeugt. Aktuelle Befunde legen nahe, dass Agency und Ownership interagieren können, dabei aber dissoziierbar bleiben, da man Agency auch über ein nicht verkörpertes Objekt erleben kann oder Ownership über ein Objekt, das sich nicht kontrollieren lässt. Das Verständnis der neurokognitiven Mechanismen, die Agency und Ownership zugrunde liegen, ist entscheidend, um die Herausforderungen des gesunden Alterns zu bewältigen. Da die sensomotorische Präzision im Laufe des Lebens abnimmt, wird die Aufrechterhaltung eines kohärenten Körper- und Kontrollgefühls zunehmend essenziell für Mobilität, die erfolgreiche Nutzung assistiver Technologien und die kognitive Resilienz im Allgemeinen. Dieser Übersichtsartikel fasst aktuelle Erkenntnisse zur Agency-Ownership-Interaktion zusammen, erörtert deren neurobiologische Grundlagen und untersucht ihr transformatives Potenzial für die Gestaltung „selbst-inklusiver“ Prothesen und Gehirn-Maschine-Schnittstellen. Abschließend skizzieren wir ein theoretisches Rahmenkonzept, das aufzeigt, wie die Optimierung dieser Prozesse funktionale Unabhängigkeit und psychisches Wohlbefinden in einer alternden Bevölkerung fördern kann.
