# Supplementary material for: The dynamic bodily self across lifespan: Agency, ownership, and the plasticity of internal body models
Source: Psychon Bull Rev. 2026 Oct 9;33(8):254. doi: 10.3758/s13423-026-03024-0 (PMC13652922; doi:10.3758/s13423-026-03024-0)
Supplement: Supplementary file 2 [file 13423_2026_3024_MOESM2_ESM.docx]

چکیده

دو جنبه بنیادی در پژوهش‌های مربوط به خودآگاهی بدنی، یعنی حس عاملیت و احساس مالکیت بدن، در سال‌های اخیر شاهد رشدی تصاعدی بوده‌اند. مقاله پیشگامانه بوتوینیک و کوهن در سال ۱۹۹۸، ابزار روان‌فیزیکی مهمی را برای مطالعه «خود بدنی» فراهم کرد: توهم دست لاستیکی؛ پدیده‌ای که در آن فرد احساس می‌کند یک شیء خارجی به بخشی از بدن او تبدیل شده است. دیدگاه‌های اخیر در حوزه کنترل حرکتی درباره احساس مالکیت بدن، بر تأثیر حس عاملیت، یعنی احساس فرد مبنی بر اینکه کنترل حرکات خود را در اختیار دارد، تأکید می‌کنند. پیشرفت‌های فناوری، توسعه پارادایم‌های آزمایشی با اعتبار بوم‌شناختی بیشتر، از جمله نسخه‌های واقعیت مجازی توهم دست لاستیکی، را امکان‌پذیر ساخته‌اند و از این طریق به گسترش درک ما از سازوکارهایی کمک کرده‌اند که مغز به‌واسطه آن‌ها احساس عاملیت و مالکیت بدن را ایجاد می‌کند.

یافته‌های اخیر نشان می‌دهند که حس عاملیت و احساس مالکیت بدن می‌توانند با یکدیگر تعامل داشته باشند، در حالی که همچنان از یکدیگر قابل تفکیک باقی می‌مانند؛ به این معنا که فرد ممکن است نسبت به شیئی که آن را به‌عنوان بخشی از بدن خود تجربه نمی‌کند، حس عاملیت داشته باشد، یا نسبت به شیئی که قادر به کنترل آن نیست، احساس مالکیت بدنی را تجربه کند. درک سازوکارهای عصبی‌ـ‌شناختی زیربنای حس عاملیت و احساس مالکیت بدن برای مواجهه با چالش‌های سالمندی سالم اهمیت اساسی دارد. با کاهش دقت حسی‌ـ‌حرکتی در طول عمر، حفظ احساسی منسجم از بدن و کنترل بر آن برای حفظ توانایی تحرک، پذیرش و استفاده موفقیت‌آمیز از فناوری‌های کمکی و تاب‌آوری شناختی کلی ضروری می‌شود.

این مقاله مروری، یافته‌های کنونی درباره تعامل میان حس عاملیت و احساس مالکیت بدن را یکپارچه می‌کند، زیرساخت‌های عصب‌زیستی این تعامل را مورد بحث قرار می‌دهد و ظرفیت تحول‌آفرین آن را برای طراحی پروتزهای «خودشمول» و رابط‌های مغز و ماشین بررسی می‌کند. در پایان، چارچوبی ارائه می‌کنیم که نشان می‌دهد چگونه بهینه‌سازی این فرایندها می‌تواند به حفظ استقلال عملکردی و بهزیستی روان‌شناختی در جمعیت سالمند کمک کند.
